# Surface Acoustic Wave Immunosensor for Detection of Botulinum Neurotoxin

**DOI:** 10.3390/s23187688

**Published:** 2023-09-06

**Authors:** Michał Grabka, Krzysztof Jasek, Zygfryd Witkiewicz

**Affiliations:** Institute of Chemistry, Faculty of Advanced Technologies and Chemistry, Military University of Technology, 00-908 Warsaw, Poland; krzysztof.jasek@wat.edu.pl (K.J.); zygfryd.witkiewicz@wat.edu.pl (Z.W.)

**Keywords:** SAW, label-free detection, botulinum neurotoxin, biological warfare agents (BWA), on-site detection of BWA, biological aerosol

## Abstract

A Love-type acoustic wave sensor (AT-cut quartz substrate, SiO_2_ guiding layer) with a center frequency of approximately 120 MHz was used to detect a simulant of pathogenic botulinum neurotoxin type A—recombinant of BoNT-A light chain—in liquid samples. The sensor was prepared by immobilizing monoclonal antibodies specific for botulinum neurotoxin via a thiol monolayer deposited on a gold substrate. Studies have shown that the sensor enables selective analyte detection within a few minutes. In addition, the sensor can be used several times (regeneration of the sensor is possible using a low pH buffer). Nevertheless, the detectability of the analyte is relatively low compared to other analytical techniques that can be used for rapid detection of botulinum neurotoxin. The obtained results confirm the operation of the proposed sensor and give hope for further development of this label-free technique for detecting botulinum neurotoxin.

## 1. Introduction

**Figure 1:**
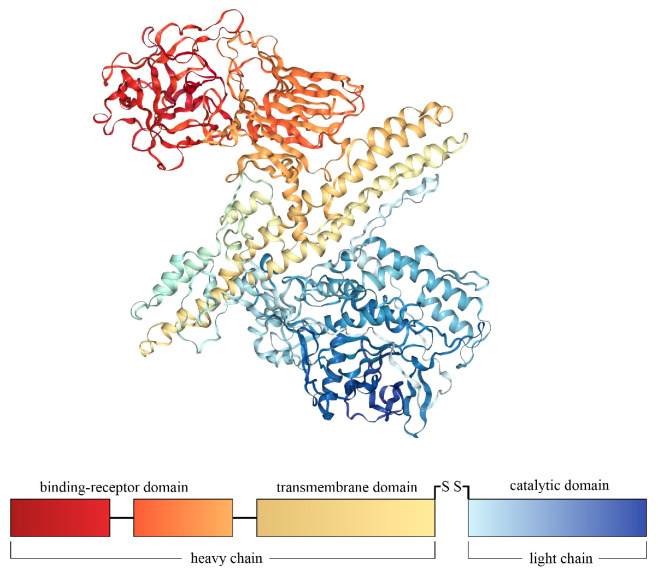
Botulinum neurotoxin type A (BoNT-A) crystal structure and schematic diagram of functional domains. The image of the crystal structure of the toxin was taken from the RCSB PDB (RCSB.org) of PBD ID 3BTA [1].

Botulinum toxin is produced, among others, by the anaerobic bacteria *Clostridium botulinum*. This toxin occurs in seven primary serotypes, of which four (BoNT-A, BoNT-B, BoNT-E, BoNT-F) cause botulism in humans, and two (BoNT-A and BoNT-B) are of exceptionally high toxicity. Botulinum neurotoxin is a protein toxin with a molecular weight of approximately 150 kDa. An active form of the toxin consists of two polypeptide chains (light chain—about 50 kDa, and heavy chain—about 100 kDa) connected by a disulfide bridge. Four functional domains can be distinguished within the toxin. Figure 1 shows the crystal structure of BoNT-A and the functional description of the domains.

The effect of the toxin is the impairment of the peripheral nervous system. The poisoning mechanism consists of the toxin’s penetration into the neuromuscular synapses, in which the neurotransmitter is acetylcholine. The heavy chain of toxin then binds specifically to receptors on the presynaptic membrane of the axon and enters it by endocytosis. Inside the cell, the light chain (catalytic domain) separates from the heavy chain and, leaving the vesicle, penetrates the cytosol. The light chain recognizes and cleaves SNARE proteins (SNARE—soluble N-ethylmaleimide-sensitive factor attachment proteins receptor), responsible for the docking and fusion of acetylcholine-filled synaptic vesicles with the presynaptic membrane. As a result of the inactivation of SNARE proteins, the release of acetylcholine by exocytosis into the synaptic cleft is inhibited. The lack of a neurotransmitter inhibits the conduction of nerve impulses and leads to flaccid paralysis.

Botulinum neurotoxin is one of the most toxic substances in the world. In the case of BoNT-A, the inhalation lethal dose LD50 is 10–13 ng/kg of body weight [2]. Comparing this value to the lethal dose of nerve chemical warfare agents (e.g., for VX, the estimated inhalation LD50 value is approximately 15 μg/kg (calculated based on [3])), it can be concluded that this neurotoxin is over a thousand times more toxic than most potent chemical agents. Botulinum neurotoxin is also classified as a biological warfare agent (category A by the Centers for Disease Control and Prevention—CDC [4]). Over the years, many countries have conducted intensive research on the production of botulinum toxin for its combat use and the most effective ways to use it on the battlefield [5,6]. An example here may be the research conducted by the Americans in Fort Detrick [7], the experiments carried out by the Japanese in Harbin (China) [8], or the work carried out after World War II by the Soviets at the Sverdlovsk facility [9]. Potentially the most likely way to use botulinum toxin is via a bio-aerosol. The toxin in this form (depending on the degree of dispersion) can move with the wind over long distances, creating an inhalation hazard for military personnel and civilians.

Currently, the detection of botulinum toxin on the battlefield is based on spectrometric methods (e.g., ultraviolet laser-induced fluorescence—UV-LIF [10]) and immunological methods (mainly lateral flow assay—LFA [11,12] or enzyme-linked immunosorbent assay—ELISA [13,14]). In the case of spectrometric methods, the undoubted advantage is the short analysis time and the possibility of detection in the stand-off mode. However, these methods are characterized by a low selectivity and are more suitable for the detection of a biological aerosol and its classification rather than for the identification of a biological agent (not to mention the identification of the toxin serotype). In the case of immunological methods, a significant advantage is a high selectivity, which allows one to determine the serotype of the toxin, which is of great importance in the possible medical treatment of people exposed to this toxin. The disadvantages of immunological methods include a much longer analysis time, the need to perform a relatively complicated analytical protocol (in the case of ELISA), and a low sensitivity (in the case of LFA) [15].

Field detection of botulinum toxin is still challenging. For this reason, research into alternative detection methods is still ongoing. Among the more interesting solutions, there are some modifications of immunological methods, such as immunological polymerase chain reaction—immuno-PCR tests (using DNA-coupled botulinum toxin antibodies [16])—or the technique of immunoassays using extrinsic immunolabels for surface-enhanced Raman spectroscopy—SERS [17].

A versatile platform for building various types of biosensors is also devices with an acoustic wave that enable conducting analyzes in the liquid phase. Devices of this type include: quartz crystal microbalance—QCM—or devices with a surface acoustic wave—SAW (Love-type SAW or shear horizontal—SH SAW). Like other immunological methods, acoustic wave biosensors use a specific antibody–antigen reaction. In this case, detecting the emerging antibody–antigen complex is different than other immunological methods. As a result of attaching the analyte to the sensor layer (e.g., utilizing immobilized antibodies), the sensor surface is loaded with additional mass, and the modulus of elasticity of the sensor layer changes. Changes in the sensor’s surface conditions affect the frequency, amplitude, and phase of the acoustic wave propagating in the underlying substrate [18]. Changes in wave parameters are the analytical signal of acoustic wave sensors. In the case of acoustic wave sensors, the detection of antigens is carried out directly (label-less). The No need to use labels simplifies the analytical procedure and significantly reduces the analysis time. This will also make it easier to implement this technique in an automated analyzer. The literature describes the use of acoustic wave sensors for the detection and determination of various biological agents, including pathogens [19,20]). In addition, multiple toxins, such as Staphylococcus aureus enterotoxin, have been detected using this type of sensor [21]. To our knowledge, however, acoustic wave sensors have not yet been used to detect botulinum neurotoxin.

This paper presents the first study’s results on using Love-type surface acoustic wave immunosensors for detecting botulinum neurotoxin type A (light chain only) in the direct assay format. The obtained results show that the detection of this analyte, which can be treated as a simulant of the pathogenic botulinum toxin, is within the scope of this analytical technique. These results constitute the proof of concept and can be used as a starting point in improving the technique and, subsequently, developing a complete device for detecting BoNT in field conditions, which is the target area of application of this technique.

## 2. Materials and Methods

### 2.1. Reagents

The following chemical reagents were used in the sensor functionalization process: 6-mercapto-1-hexanol (95%, Sigma-Aldrich, St. Louis, MO, USA, C6OH), 11-mercaptoundecanoic acid (95%, Sigma-Aldrich, MUA), N-hydrosuccinimide (98%, Sigma-Aldrich, NHS), N-(3-dimethylaminopropyl)-N′-ethylcarbodiimide (97%, Sigma-Aldrich, EDC), ethylamine (70% in water, Acros Organics, EtNH_2_).

The following bioreagents were used in the work: anti-BoNT-A monoclonal antibodies specific for toxin light chain (R&D Systems, Minneapolis, MN, USA, catalog number: MAB4489, MAb), recombinant of botulinum neurotoxin type A light chain (R&D Systems, catalog number: 4489-ZN, BoNT-A LC), and bovine serum albumin (98%, Sigma-Aldrich, BSA).

The following buffer solutions were used in work: sterile and filtered phosphate buffer saline—reconstitution/coating/running buffer (R&D Systems, catalog number: DY006, PBS), 2-(N-morpholino)ethanesulfonic acid (MES) buffer (0.1 M, pH = 6.20)—used in EDC/NHS activation step, sodium borate buffer (0.1 M, pH = 8.75)—used in ethylamine blocking step, PSB with 0.05% TWEEN^®^ 20 Detergent (BioVison, Waltham, MA, USA, catalog number: 2130, PBST)—wash buffer, Glycine-HCl buffer (0.1 M, pH = 2.5, Gly-HCl)—regeneration buffer. BoNT-A LC solutions in PBS were used to test sensors’ response.

### 2.2. Instrumentation

**Figure 2:**
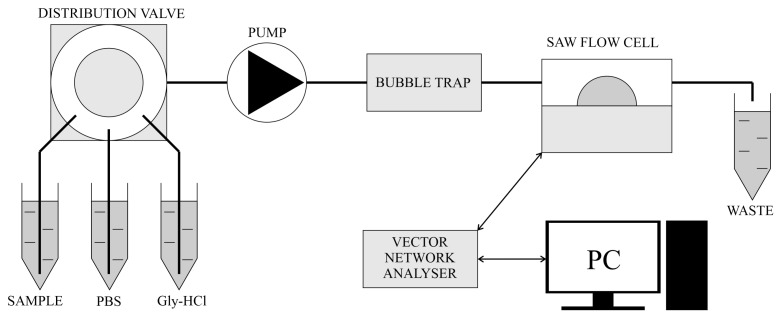
Scheme of the measurement system used in the work.

Figure 2 shows a diagram of the measuring system used in the measurements.

The microfluidic system consisted of containers (Eppendorf type) with running buffer, sample and regeneration buffer, distribution valve (Rheodyne, Rohnert Park, CA, USA, model MXT715-0000), in-line microfluidic bubble trap (Elveflow), liquid flow cell for Love-type SAW delay lines (AWS, Seattle, WA, USA, model CLS00028A), peristaltic pump (LeadFluid, Baoding, China, model BT102S), and waste container. All measurements were made at an ambient temperature of approximately 25 °C. 

This work used Love-type SAW delay lines with a resonant frequency of about 120 MHz (AWSensors, Valencia, Spain, model SNS000069A). The SAW devices were manufactured on a 17.0 by 8.4 mm AT-cut quartz substrate with a thickness of 0.35 mm. Two groups of aluminum interdigitated electrodes (interdigitated transducers—IDTs) were deposited on the surface of the substrate (number of pairs of electrodes N = 100). The periodicity of the IDTs was 40 µm and the acoustic aperture (distance between groups of electrodes) was 3.5 mm. A 3 μm thick SiO_2_ guiding layer was applied on the substrate using plasma-enhanced chemical vapor deposition—PECVD. A layer of gold (50 nm thick, on 10 nm Cr) was applied to the surface of the guiding layer, which was further functionalized as described below.

A vector network analyzer (mRS, Warrendale, PA, USA, model miniVNA PRO^2^) was used to monitor the sensor signal in the cell. The signal was monitored and recorded using a self-written application for a PC. The application allowed for monitoring of the sensor’s frequency changes, attenuation, and phase shift.

## 3. Results and Discussion

### 3.1. Functionalization of SAW Devices

**Figure 3:**
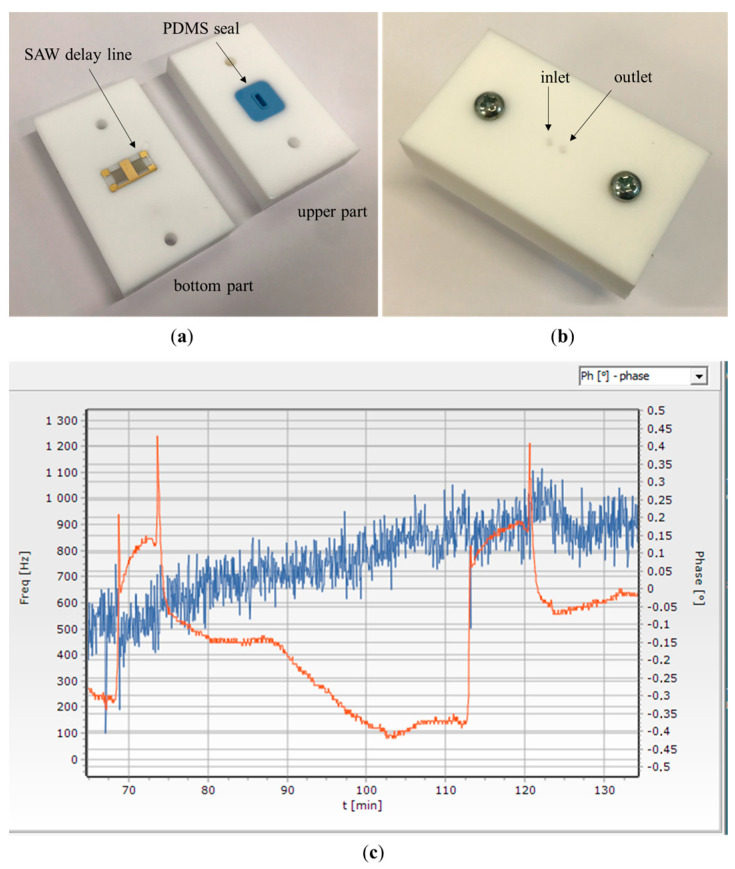
Home-made functionalization cell in (**a**) disassembled and (**b**) folded state. (**c**) Screenshot of the control application during measurement with BoNT-A LC (visible changes in phase—red line, and frequency—blue line, due to BoNT-A LC dosing and sensor regeneration).

Each SAW delay line was cleaned with Piranha solution (H_2_SO_4_/H_2_O_2_, 3:1 by volume) by immersing the whole device in this solution for 2 min. Then, a device was washed with deionized water and ethanol and dried in a stream of nitrogen. Immediately after cleaning, the device was immersed in an alcoholic solution of thiols. A mixed thiol monolayer was chosen based on literature data [22]. The solution of thiols in ethanol with a total molar concentration of 10 mM/dm^3^ contained MUA and C6OH (molar proportions 1:3). The device was left in solution for 24 h at room temperature (RT) with gentle stirring. After this time, the device was washed with ethanol, dried in a nitrogen flow, and immediately transferred to a home-made functionalization cell (Figure 3).

**Figure 4:**
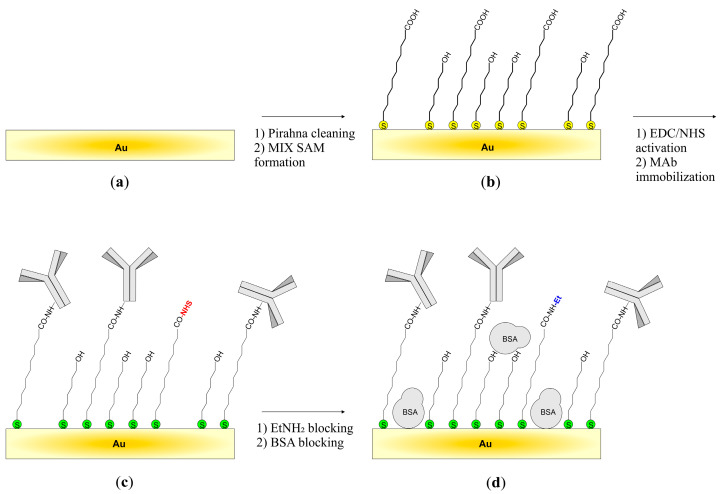
Sensor fabrication steps: (**a**) substrate preparation, (**b**) mixed thiols self-assembled monolayer—MIX SAM formation, (**c**) MAb immobilization, (**d**) layer blocking to minimize non-selective interactions.

The cell was made of Teflon^®^ and enabled the functionalization of the sensor by isolating the sensitive area (approximately 16 mm^2^) from the rest of the device containing the electrodes. As a result, aqueous protein solutions washed only the sensitive area. In addition, the sensor was not exposed to air between successive functionalization steps. Furthermore, the small working volume of the cell (0.1 mL) minimized the consumption of reagents. After placing the SAW device in the functionalization cell, the acid residues of the thiol monolayer were activated to a more reactive form (N-hydroxy-succinimide ester). For this purpose, a solution with a concentration of 0.1 M NHS and 0.1 M EDC was prepared in MES buffer (pH = 6.20). Immediately after preparing the solution, it was introduced into the functionalization cell and left for 1 h at RT. After this time, the cell was washed several times with MES buffer, and then with sterile PBS. In the next step, a solution of MAb in sterile PBS with a 25 μg/mL protein concentration was introduced into the cell. The cell was left for 4 h at RT with gentle stirring. After this time, the cell was rinsed with PBS. In the next step, the remaining N-hydroxy-succinimide ester groups were deactivated. For this purpose, the cell was washed with borate buffer (pH = 8.75), and then an ethylamine solution (1 M in borate buffer) was introduced into the cell. The cell was left for 1 h at RT and then rinsed with borate buffer. After that, non-selective sorption sites on the sensor surface were blocked with BSA solution. The cell was rinsed with PBS buffer, and then a BSA solution in PBS with a protein concentration of 1% by weight was introduced into the cell and left for 1 h in RT. After this time, the sensor was removed from the functionalization cell and intensively rinsed with PBST. Finally, the sensor was rinsed with deionized water and dried in a stream of nitrogen. Afterward, the sensor was packed under nitrogen in a sealed vessel and stored at 4 °C. The sensor preparation process is schematically shown in Figure 4.

**Figure 5:**
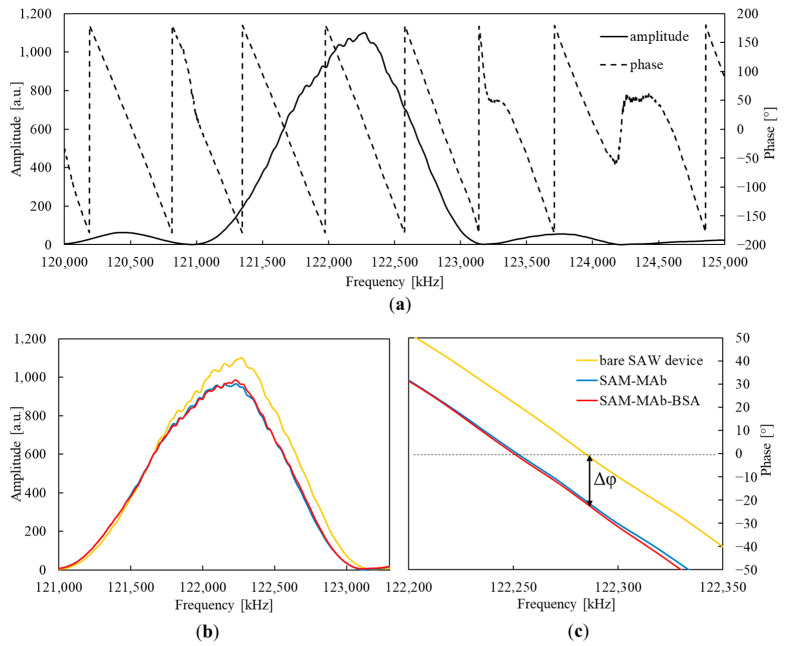
(**a**) Exemplary frequency characteristics of a bare SAW device recorded in air. (**b**) Amplitude and (**c**) phase characteristics of the SAW device recorded in air after successive stages of functionalization (color markings are common to (**b**,**c**)).

The individual steps of functionalization were monitored by measuring the electrical parameters of the SAW devices. Figure 5 shows an exemplary sensor’s amplitude and phase characteristics before and after functionalization.

As seen in Figure 5b,c, the functionalization caused both amplitude and phase characteristics changes. However, changes resulting from forming a thiol monolayer are not observed in the characteristics (the plot is not shown in Figure 5). The deposition of MAb on the EDC/NHS-activated thiol layer causes a significant increase in attenuation and a shift in the phase characteristic by an angle of about −20°. Subsequent blocking with ethanolamine and BSA induced only slight changes in the phase characteristics. The final shift in the phase characteristic is marked in Figure 5c as Δφ.

The measurements of the characteristics were repeated after a series of tests lasting several days, during which the sensors were repeatedly exposed to the analyte solution, regenerated, and rinsed with the running buffer. There was no shift in the characteristics by an angle greater than the estimated measurement error (the maximum error of the angle measurement resulting from the positioning of the SAW sensor in the flow cell is equal to 3°), which proves the permanent immobilization of proteins on the surface of the SAW device.

The repeatability of sensor functionalization was also estimated. The average value of Δφ obtained for four devices functionalized according to the procedure described above is equal to −22.3 ± 2.4°.

### 3.2. BoNT-A LC Detection

The prepared sensors were tested in the measurement system described in the Section 2.2. After mounting the sensor in the measuring chamber, it was rinsed with a running buffer for 3 h at a flow rate of 40 µL/min before starting the measurements. The sensors were tested within a week of manufacturing (functionalization) and stored at 4 °C between measurements. A typical sensor measurement cycle was as follows:

- Sensor rinsing with PBS (running buffer) at 40 μL/min (baseline stabilization);
- Sample dosing (solution of a specific protein in PBS) at 40 μL/min;
- Sensor rinsing with PBS at 40 μL/min;
- Regeneration of the sensor with the Gly-HCl buffer at 100 μL/min flow rate;
- Sensor rinsing with PBS at 40 µL/min (baseline stabilization).

Immediately before each measurement, fresh protein samples in PBS were prepared from their stock solutions. 

**Figure 6:**
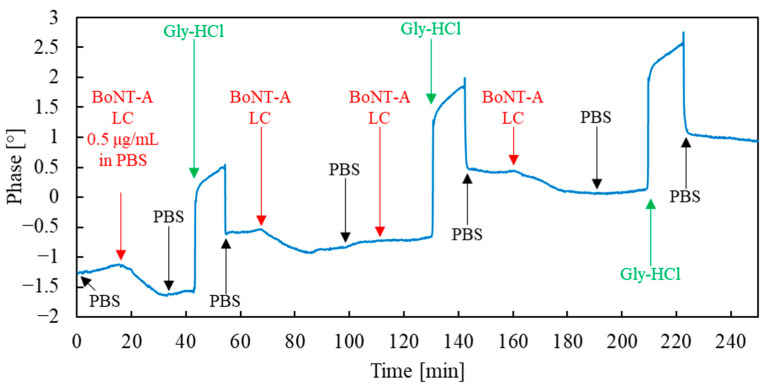
Phase versus time plot of sensor recorded during three subsequent measurement cycles. The initial dosing times of individual solutions are marked with arrows (the times have been increased by the time resulting from the dead volume of the microfluidic system at a given flow rate). The beginning of a given solution dosing also means the end of the dosing of the previous solution. Black color indicates the flow of running buffer PBS, red—BoNT-A LC dosing in PBS at a concentration of 0.5 ug/mL, and green—Gly-HCl regeneration buffer flow.

Figure 6 shows three consecutive sensor measurement cycles, including exposure to BoNT-A LC solution, PBS rinsing, and sensor regeneration.

The measurement system with a vector analyzer, apart from the phase (SAW phase at a fixed frequency), allowed for the recording of the amplitude and frequency of the resonance peak. However, since the best signal-to-noise ratio characterized the phase signal, further analysis was based on this signal (similarly as in the work [23]). The analytical signal was adopted as the phase shift between the beginning of toxin dosing and the following steady-state value of phase (or, in a transient state—value of phase after 10 min from the start of toxin dosing).

As can be seen, in three consecutive measurement cycles, the sensor reacted to the presence of the analyte by clearly changing the SAW phase. It can also be seen that the phase changes corresponding to subsequent analyte dosing decrease with each successive cycle. 

In Figure 6, the 60 to 120 min interval requires additional comment. From 68 to 100 min, a phase change resulting from the sorption of the toxin is visible. The flow of the toxin solution is then turned off, and the sensor is rinsed with a clean buffer solution (from 100 min). Turning off the toxin does not return the phase to its original value. From 110 min, the toxin flow was turned back on, but this did not result in any further phase change this time. This situation may result from forming a stable antibody–antigen complex during first exposure to the toxin (from 68 to 100 min). The sensor signal is saturated after the depletion of free sorption sites (accessible epitopes of antibodies). Washing the sensor with a clean buffer does not break the complex; therefore, dosing a new portion of the toxin solution does not generate a sensor signal. Detection of the toxin is possible only after regeneration of the sensor with Gly-HCl (breaking the antibody–antigen complex at low pH).

The sensors were tested at 0.1, 0.5, and 1 μg/mL analyte concentrations. However, the sensor signal for the 0.1 μg/mL concentration was characterized by a small amplitude and was difficult to distinguish from a short-term baseline drift. For this reason, these results were omitted from further consideration. Table 1 compares the average sensor response values obtained for BoNT-A LC concentrations of 0.5 μg/mL and 1 μg/mL recorded during their first use of sensors (first measurement cycle).

The maximum phase shifts obtained for both tested concentrations are about 0.5° (no concentration dependence is observed here). These values are relatively small, but they clearly distinguish the signal from the baseline drift. However, based on the obtained data, it is impossible to draw clear conclusions about the concentration dependence of the sensor signal in the transient state (signals recorded after 10 min).

The average response time of the sensors was 19 ± 1.1 min (from the start of analyte dosing to the steady-state value of the signal). Despite this, the signal value allowing for an undisputed determination of the detection of the analyte was achieved after less than 10 min.

Table 2 presents the botulinum toxin detection limits and response times achieved with other techniques that can be used in devices for the non-laboratory and rapid detection of botulinum toxin.

In our study, the lowest empirically detected concentration of analyte was 0.5 μg/mL, which is much higher than the LODs of other analytical techniques reported in the literature. Note, however, that this value does not actually represent the LOD (we have omitted estimating the LOD due to the presence of significant short-term drift in our measurement system). Based on this data, it can be concluded that, in its current form, the BoNT detection technique using SAW sensors does not offer a satisfactory detectability of this analyte.

There are many potential ways to improve the detectability of botulinum toxin using the acoustic wave sensor. The most important ones include applying a more sophisticated immunoassay format (e.g., a sandwich assay using a secondary antibody conjugated with gold NPs [31]) or the use of sensor layers with an extensive, porous surface (e.g., immobilized antibodies on a layer of porous gold [32]). 

This paper presents toxin detection in the most straightforward, direct system—direct assay format. In addition, the sensor layer was deposited on a flat, polished gold substrate. It is also worth mentioning that only a fragment of the active form of botulinum toxin was used in this work—a light chain with a mass of about 50 kDa. In the case of a pathogenic toxin, it is about 150 kDa, which would positively impact the detection of this substance since SAW transduces work to a large extent as a gravimetric sensor.

By comparing the values of response times from Table 2, it can be concluded that the Love-type acoustic wave sensor allows for obtaining results in a short time, comparable to the time of analysis using LFA tests.

### 3.3. Selectivity of Sensor

Analyte sorption can occur by the selective formation of an antibody–antigen complex or by non-selective interactions related to Coulomb intermolecular interactions between the antigen and the sensor substrate. Additional measurements were carried out to verify the mechanism of analyte sorption in the case of the prepared sensors. 

**Figure 7:**
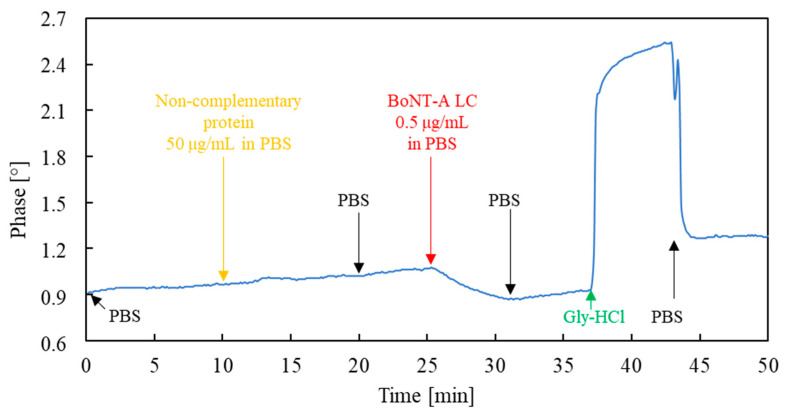
Phase versus time plot of the sensor exposed to a non-complementary protein solution to the immobilized antibodies and then to a BoNT-A LC solution.

A protein solution in PBS that was not complementary to the immobilized antibody was used for this test. In this case, a solution of a BoNT-A light chain antibody, previously used in the sensor preparation process, was used. The protein concentration in the sample was 100-fold higher than the concentration of the analyte during the measurement. The phase versus time plot of the sensor recorded during the selectivity test is presented in Figure 7.

As can be seen from the figure above, the sensor exposed to the BoNT-A light chain antibody solution (from 10 to 20 min) did not show a signal distinguishable from the baseline drift despite the high concentration of this non-specific protein in the sample. Exposure to this protein also did not affect the subsequent detection of BoNT-A LC. 

Although the presence of a BoNT-A light chain antibody in the actual environmental sample is unlikely, and it cannot be treated as a possible interferent, this experiment aimed only to confirm the mechanism of selective antigen binding. A BoNT-A light chain antibody does not form an antibody–antigen complex with itself. Nevertheless, it is an adhesive protein, and, like any protein, it can be sorbed by non-specific Coulomb interactions. The lack of a visible signal (10 to 20 min) proves that non-selective interactions play little role in signal generation. At the same time, the noticeable signal value after subsequent exposure to the analyte (BoNT-A LC) indicates that it is sorbed by selective antibody–antigen interactions.

**Figure 8:**
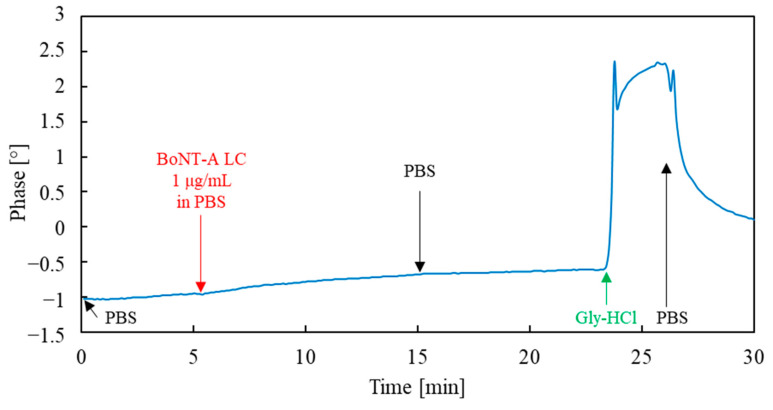
Phase versus time plot of the sensor with immobilized BSA exposed to BoNT-A LC.

An additional experiment was also carried out to verify the sorption mechanism of the analyte itself, i.e., BoNT-A LC. For this purpose, a sensor functionalized with the BSA protein, instead of the BoNT-A light chain antibody, was prepared (the rest of the sensor preparation procedure was identical as in the case of the MAb sensor). The BSA sensor was tested with a 1 μg/mL BoNT-A LC solution in PBS. The phase versus time plot of the sensor recorded during this experiment is shown in Figure 8.

As can be seen from Figure 8, exposure of the BSA sensor to the analyte did not produce a significant signal. The result of this experiment is further evidence that non-selective interactions have a small contribution to the sorption of BoNT-A LC, and the considerable signal values observed during MAb sensor measurements resulted from sorption by selective immunological interactions.

### 3.4. Reusability of Sensor

**Figure 9:**
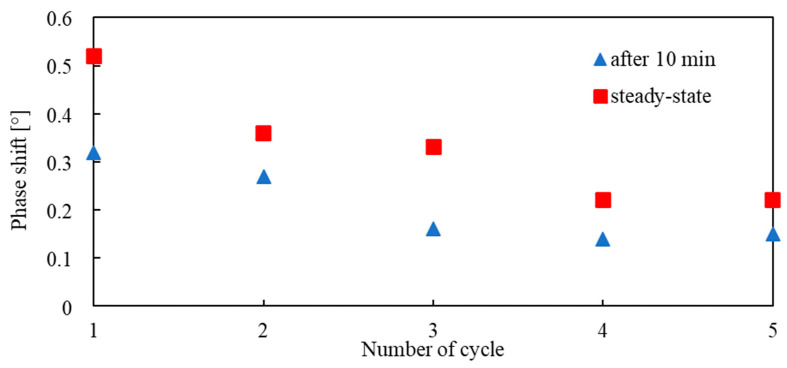
Sensor response values to the solution of BoNT-A LC in PBS at a concentration of 0.5 μg/mL recorded 10 min after starting dosing (blue triangles) and steady-state values (red squares) in the successive five measurement cycles of the sensor.

As noted earlier, the sensor can be regenerated after exposure to BoNT-A LC. Breaking the antibody–antigen complex is possible by drastically lowering the pH. In this case, a Gly-HCl buffer with a pH of 2.5 at 25 °C was used. However, the regeneration of the sensor reduces the signal value with subsequent exposures to the analyte (also visible in Figure 6). The influence of the repeated regeneration of the sensor on its properties was investigated as part of the work. The results are presented in Figure 9.

As can be seen from the figure above, after four measurement cycles, the sensor signal decreased by approximately 60% compared to the first cycle. In the fifth cycle, a further decline was no longer observed. 

The ability to regenerate the sensor is a desirable feature, but even a sensor that allows for a single determination of analytes can also fulfill its role. A significant decrease in detection, but one that does not render the sensor useless, is observed in the case of prepared sensors. The relatively high reusability is probably due to the method of MAb immobilization. In this case, the antibodies were covalently bound to the thiol monolayer via a peptide bond. An alternative solution is to use an interface from another protein, e.g., protein A, whose task is to orient antibodies better and improve epitope accessibility. As a result, a unit area of the sensor layer can bind more antigen molecules, and the sensor signal is amplified. Such a protein is covalently bound to the thiol monolayer, while MAb attachment is based on non-covalent interactions. As a result, during the sensor regeneration, the antibody may be washed out in addition to the antigen. Of course, this causes a radical decrease in the sensor’s sensitivity in subsequent measurement cycles.

## 4. Conclusions

The proposed immunosensor allows for the selective detection of BoNT-A LC in liquid samples. The analysis time is short compared to other analytical techniques and allows for near real time monitoring of the presence of the analyte. However, at the current stage of development, the analyte detectability is much worse than in the case of the previously described methods (see Table 2), which is the main limitation of the developed immunosensor. To enable further development of this sensor, it will be necessary to improve the detectability of the analyte.

A potential future application area of the described technique may be the field detection of botulinum toxin used as a weapon of mass destruction in the form of a biological aerosol. Building a complete analyzer that could take the form of a hand-held or vehicle-mounted device (including onboard unmanned aerial vehicles—UAVs analyzer) will require developing an aerosol sampling system. Such a system should collect aerosol particles into a concentrated liquid sample for further analysis using the SAW sensor. The sample collection system could use air filtration and sample elution from the filter similar to the commercially available BioCapture z720 (manufactured by BioFlyte [33]), or a cyclone [34].

In our opinion, due to low equipment requirements, high potential for miniaturization, and the possibility of constructing devices that operate automatically, the technique of immunosensors based on acoustic wave transducers has a good chance of practical application in the on-site detection of botulinum toxin, but only after addressing the issue of the low detectability of the analyte.

## Figures and Tables

**Table 1:** Average sensor response values (phase shifts) recorded during the first exposition of sensors to BoNT-A LC solution.

BoNT-A LC Concentration [μg/mL]	Phase Shift after 10 min [°]	Phase Shift in Steady State [°]	Number of Sensors Tested
0.5	0.29 ± 0.04	0.51 ± 0.07	3
1	0.32 ± 0.06	0.51 ± 0.06	3

**Table 2:** A comparison of some immunological analytical techniques potentially useful in non-laboratory rapid detection of botulinum toxin.

Analytical Technique	Limit of Detection—LOD	Time of Analysis	Reference
ELISA (enzyme-linked immunosorbent assay)	5 pg/mL–2 ng/mL	5–6 h	[24,25]
LFA (lateral flow assay)	5–50 ng/mL	15 min	[11,12,26]
Flow cytometry	50 pg/mL–20 ng/mL	4 h	[27,28,29,30]
Love-type acoustic wave sensor	500 ng/mL ^1^	19 min	this work

^1^ In fact, this value does not actually represent the LOD but is the lowest test concentration determined in the measurements.

## Data Availability

The data presented in this study are available on request from the corresponding author.
